# Chronic Headache Associated with Pelvic Disease

**Published:** 1907-01

**Authors:** 


### CHRONIC HEADACHE ASSOCIATED WITH PELVIC DISEASE.

(*Journal American Madical Association*, November 10, 1906).

In an article on chronic headache associated with pelvic disease, Dr. F. H. Davenport, of Boston, draws the following conclusions from the analysis from one thousand cases taken from his own practice. In the one thousand cases he finds chronic headache mentioned 98 times, constituting ten per cent., approximately, of the cases, which is a much smaller number than one would expect. He divides these cases into two groups: first, those due to menstrual disturbances. Of the first group he says as follows:

Taking the first group, I find that 30 cases, or about one-third, are directly associated, as regards time, with menstrual disorders, and the one abnormality which is constant in these cases is a diminuation of the amount of blood lost, either from scanty flow with the regular periods, or from delayed menstruation, the interval being more than four weeks, or from both irregular and scanty menstruation combined. In many of these cases menstruation occurred very irregularly, from five weeks to two or three months between the periods.

The most common type of headache found in these cases comes on a few days before the flow occurs, lasting from one day to several, and ceasing promptly as soon as the flow is established. The headache usually precedes the period, but in a few cases in which the flow is fairly regular, but scanty, the headache appears after the period. This type frequently accompanies chronic metritis, when tissue changes have taken place in the uterus and the organ has become cirrhotic. Headaches occurring during the flow are very rare.

The explanation of this form of headache is obvious. There is a hindrance to the normal free and sufficient escape of blood, and as a result of pelvic congestion. This interference with the pelvic circulation shows itself by disturbances elsewhere and the headache is a manifestation of this.

The treatment for this variety of headache consists, first, in general measures to improve the patient's health with the hope that there will be coincidentally a change for the better in the rhythm and amount of the menstrual flow. As a rule, these patients are delicate, weak, anemic, and anything which will make them stronger physically is of value. These measures readily suggest themselves and are those which are usually employed in such cases without reference to the particular cause of the trouble or the special form in which the symptoms manifest themselves. The most important are improving the digestion by insisting on as much food as can be assimilated and of a quality which will not tax the digestive organs. Regular and suitable exercise to improve the muscular tone is of great importance. Freedom from mental strain, as study in the case of young girls and social dissipation in those older, should be insisted on.

In other words, measures which will make blood, improve its circulation and steady the nerves will tend to regulate this function. For this purpose drugs are an adjunct, but should not be depended on alone. The most important are iron and manganese, which physiologically tend to improve the quality of the blood Hand in hand with these general measures and for the relief of the headache itself, local treatment should be employed, and in this way much more direct and quick improvement can be attained. If Nature does not provide relief by a prompt escape of a sufficient amount of blood, local depletion should be employed.

Of the various methods suggested for this purpose there is none which is so simple in its application and so efficient in its results as the use of wool tampons soaked in glyerin. As soon as the slightest suspicion of headache makes its appearane, or even anticipating this by a day or two, such tampons should be inserted into the vagina and allowed to remain for two days. The result will be a profuse watery discharge of blood serum which relieves the engorged pelvic blood vessels and equalizes the circulation. At the end of the second day the tampon should be removed and a second substituted. These should be applied every second day until the flow appears.

It is not an uncommon occurrence for the patient to experience marked relief within a few hours after the first treatment, and the headache to be kept in check during the whole premenstrual epoch. If repeated before every menstruation for several months, the period of headache is shortened and in many cases a cure is effected. In the cases in which the headache follows instead of precedes the menstruation, the same treatment should be instituted as soon as the period is over and kept up for several days.

Of the second group, namely, those due to neurasthenic conditions, he draws the following conclusions:

1. Chronic headache associated with pelvic disease is accidental, except when it is the most marked symptom or irregular or scanty menstruation.

2. In other pelvic disorders, it is usually an expression of the neurasthenic ocndition of which the pelvic lesion is merel yone factor or accessory predisposing cause.

3. Treatment for the first class of cases consists of general building up measures and local depletion.

4. Treatment of the second class consists in the rectification of existing local trouble unless especially in contraindicated, but the main reliance is to be placed on prolonged and systematic treatment of the neurasthenia.—*Annals of Gyn. and Ped.*, December, 1906.

